# Placement of Unilateral Cortical Bone Trajectory Screws in Previously Instrumented Pedicle without Removal of Existing Hardware for Adjacent Segment Disease

**DOI:** 10.1155/2021/9994539

**Published:** 2021-11-09

**Authors:** Rojeh Melikian, Sofia Yeremian

**Affiliations:** ^1^DISC Sports & Spine Center, 13160 Mindanao Way, Suite 300, Marina del Rey, CA 90292, USA; ^2^Department of Molecular Cell and Developmental Biology, University of California, Los Angeles, USA

## Abstract

Adjacent segment disease (ASD) in the lumbar spine is a possible consequence in segments adjacent to a fusion. As the number of lumbar fusions in the United States increases, the rates of ASD will continue to climb. There are several treatment options for ASD with open decompression and extension of the fusion being common. However, need for exposure and removal of existing instrumentation can lead to increased operative times resulting in increasing blood loss and infection risk. The purpose of this paper is to describe a case report for unilateral cortical trajectory screw instrumentation, allowing for posterior instrumentation without having to remove the existing pedicle screws in the setting of ASD. Our technique can be done with standard c-arm fluoroscopy without the need for navigation.

## 1. Introduction

Adjacent segment disease (ASD) in the lumbar spine is a possible complication in segments adjacent to a lumbar fusion. As the number of lumbar fusions performed in the United States has increased, the rates of ASD have also climbed [1–5]. ASD can occur as a result of the normal degenerative process; however, there is evidence to suggest a solid fusion can accelerate the degenerative process in adjacent levels [6]. This contribution is likely due to an increase in range of motion and stress at the upper adjacent level [7]. With an increasingly elderly population, spinal fusions are increasing, and as a result, so are surgeries for ASD [8, 9]. When conservative measures fail to help with ASD, the most common surgical approach is an open posterior decompression and extension of the instrumented fusion [10, 11]. Exposure of all previous pedicle screws or having to cut the rod in between screws to allow for removal of the existing instrumentation can result in increased rates of complications such as blood loss and site infections due to the increased operative time [12–14]. An additional complicating factor is hardware for which no implant records are available and the type of pedicle screw instrumentation is unknown. This can result in an intraoperative situation requiring screw removal sets and the possibility of proprietary screw drivers for which there are no attachments available.

One alternative is stand-alone anterior or lateral lumbar interbody fusion [15]; however, not all patients may be candidates for stand-alone interbody fusion. In those patients where it is felt posterior fixation should be added to their interbody fusion, the cortical bone trajectory (CBT) screw allows an alternative to pedicle screw removal and has been shown biomechanically to be superior to pedicle screws [16–18]. The purpose of this paper is to describe a case report for unilateral cortical screw instrumentation without having to remove the existing pedicle screw, thus not only allowing for posterior instrumentation after interbody fusion for ASD but also avoiding the need for extensive dissection to expose the existing pedicle screws and any attempt at removing previous hardware removal. Our technique can be done with standard c-arm fluoroscopy without the need for navigation.

## 2. Case Presentation

**Figure 1:**
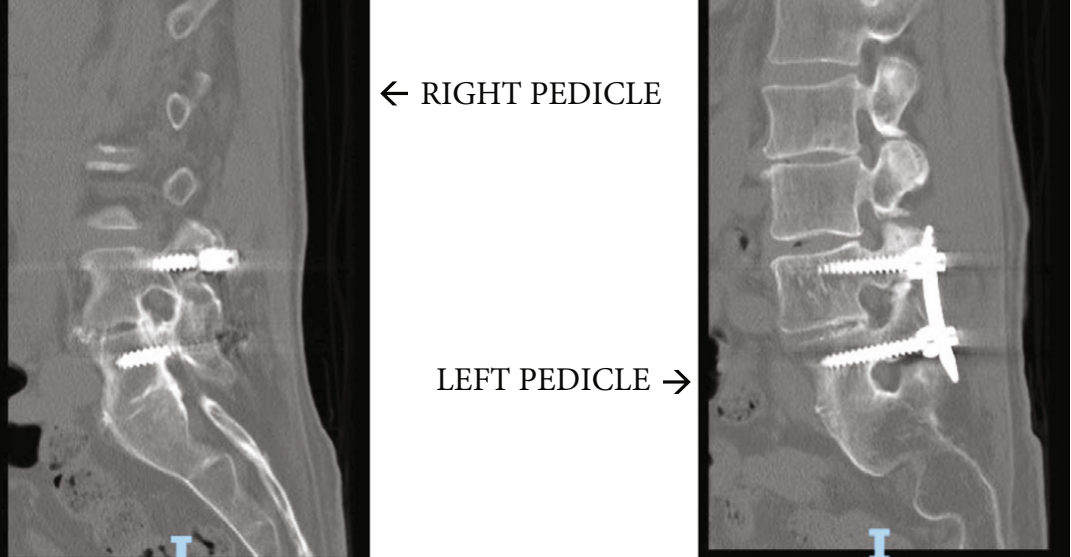


Our patient is a 77-year-old man with a previous history of L4-5 transforaminal interbody fusion (TLIF) and posterior instrumentation with pedicle screws done in 2007 at another institution. He presented to our office with complaints of recurrent low back and right-sided buttock and lateral leg pain for months. His preoperative MRI demonstrated a right-sided facet cyst at L3-4 along with lateral recess stenosis. Transforaminal epidural steroid injections only provided temporary relief, and so surgical intervention was offered. Preoperative CT scan (Figure 1) showed an adequate amount of space beneath the right-sided L4 pedicle screw for placement of a 5.5 mm cortical screw; however, the left-sided residual pedicle space was much smaller. After shared decision-making, we decided on proceeding with adjacent segment fusion and decompression. The patient was taken to the operating room, and after successful completion of the lateral interbody fusion, he was positioned prone on the Jackson table. After exposure and localization of the appropriate level, the following technique was utilized for cortical screw instrumentation.

**Figure 2:**
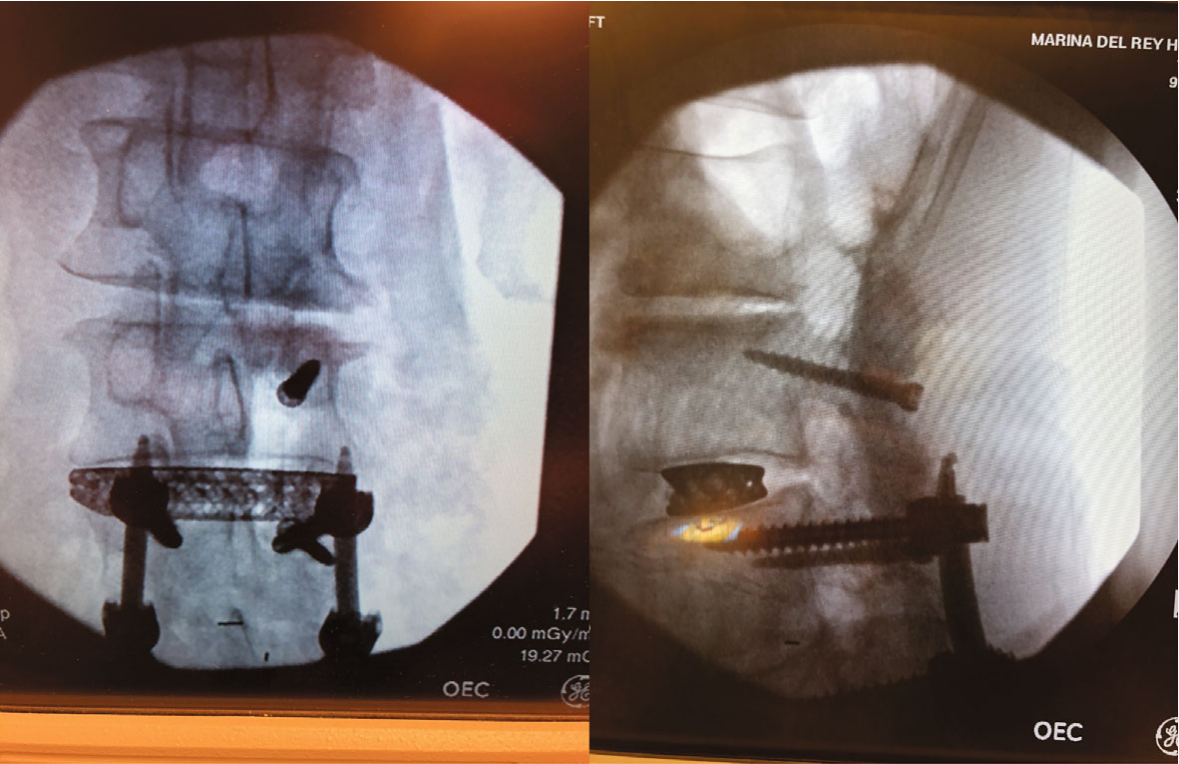


**Figure 3:**
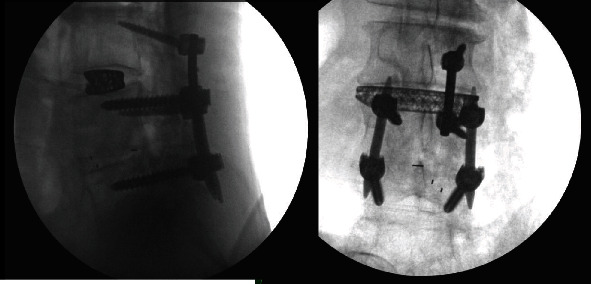


**Figure 4:**
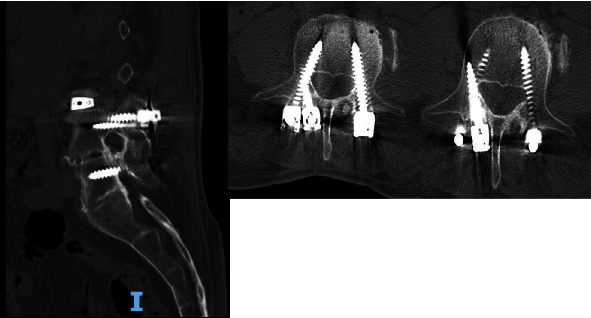


**Figure 5:**
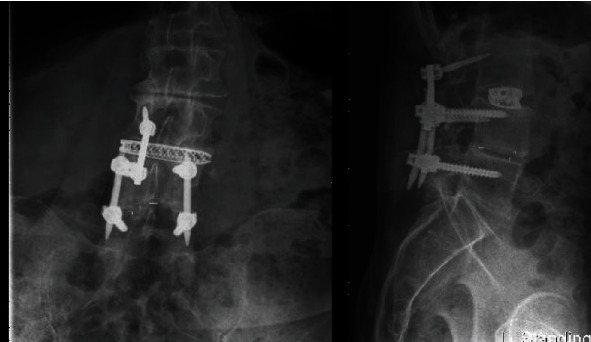


Intraoperatively, exposure is only needed laterally to the level of the pars at both levels and no further. There is no need for wide exposure beyond the pars laterally. In fact, the previous instrumentation was not seen within the operative bed. The C-arm was then brought in, and a perfect AP X-ray of the L4 pedicle was obtained. The start site for the cortical screw in terms of medial to lateral start site is then marked and the cranial-caudal start site estimated as well. Lateral C-arm X-ray is then used to confirm the cranial-caudal start site and angle. Based on the preoperative CT scan, we aimed to be just distal to the existing pedicle screw. The pedicle is then drilled aiming parallel to the pedicle screw on the lateral view but heading out slightly laterally on the AP. This is slightly different from the description of standard cortical screws where the screw is aimed cranially as the existing pedicle screw has to be adjusted for. The walls are then probed and tapped, and the appropriate screw was inserted (Figure 2). The L3 screw was then inserted using the standard cortical screw technique. The insertional torque on both screws was excellent, and so, the contralateral side was not attempted given how little room there was underneath the other pedicle screw. The screw shanks were placed in without the heads so an ipsilateral decompression could be performed without interference and the facet cyst excised. The heads were then placed on the screws, and appropriate size rod was placed (Figure 3). Set screws were then placed and final tightened, and the closure of the wound then proceeded in standard fashion. EBL for the case was 50 cc. Postoperatively, the patient's radicular symptoms were resolved, and the patient was discharged home on POD#1. Postoperative CT scan demonstrates placement of both screws inside the L4 pedicle (Figure 4). At last, postoperative follow-up at 22 months, the patient remained asymptomatic and was doing well (Figure 5).

## 3. Discussion

Adjacent segment degeneration and symptomatic disease are a potential consequence of lumbar fusion surgery. The potential mechanisms of ASD development have been suggested to be increased biomechanical stress and motion at adjacent levels, particularly, the level superior to the fusion [19, 20]. There are several treatment options of ASD with extension of the fusion being a common solution [21, 22]. Previous studies have examined stand-alone lateral interbody fusions and compared them with open posterior decompression and fusion as well [15]. However, more recent case series have looked at the feasibility of using cortical bone trajectory screws to instrument the same level as the existing pedicle screw without the need for screw removal using navigation [23]. This is advantageous in situations where patients require an open decompression and are not candidates for stand-alone lateral interbody fusion.

Cortical bone trajectory screws were first described in the lumbar spine in 2009 by Santoni et al. [24]. The placement and trajectory of this screw maximize thread contact with high-density bone and can be used to achieve contact with four bony cortices [25]. They have been shown to have 30% increased uniaxial pullout strength as compared to traditional pedicle screws and given their juxtaposition to higher density bone and are even more desirable in cases of osteoporosis [24, 26] The advantage of using cortical bone trajectory screws in adjacent segment disease is the limited dissection needed to expose the start site for the screws as well as avoidance of removing existing hardware. Previous studies have been able to show success using CT-guided navigation to place cortical screws into pedicles with existing pedicle screws [23]. We present here, to our knowledge, the first described case of C-arm guided, unilateral cortical screw instrumentation for the treatment of ASD in cases where only one pedicle has enough room to allow for simultaneous placement of a pedicle and cortical screw. This allows for screw placement to be done in settings where intraoperative navigation is unavailable and also serves as proof of concept for unilateral instrumentation with cortical screws in the setting of adjacent segment disease. Further studies need to be done to address the biomechanics of this approach, and it should be used cautiously in patients with osteoporosis. Furthermore, prospective data with larger numbers of patients with patient reported questionnaires would be desirable to validate the outcome of these patients.

## Data Availability

All references can be found on https://pubmed.ncbi.nlm.nih.gov/
